# Highly sensitive label-free in vitro detection of aflatoxin B1 in an aptamer assay using optical planar waveguide operating as a polarization interferometer

**DOI:** 10.1007/s00216-019-02033-4

**Published:** 2019-08-07

**Authors:** Ali Al-Jawdah, Alexei Nabok, Hisham Abu-Ali, Gaelle Catanante, Jean-Louis Marty, Andras Szekacs

**Affiliations:** 1 Materials and Engineering Research Institute, Sheffield Hallam University, City Campus, Sheffield, S1 1WB UK; 2 Department of Biochemistry and Molecular Biology, University of Perpignan, 66860 Perpignan, France; 3 Agro-Environmental Research Institute, NARIC, Budapest, 2100 Hungary

**Keywords:** Optical planar waveguide, Polarization interferometer, Refractive index sensitivity, Aptamer, Aflatoxin B1

## Abstract

This work reports on further development of an optical biosensor for the in vitro detection of mycotoxins (in particular, aflatoxin B1) using a highly sensitive planar waveguide transducer in combination with a highly specific aptamer bioreceptor. This sensor is built on a SiO_2_–Si_3_N_4_–SiO_2_ optical planar waveguide (OPW) operating as a polarization interferometer (PI), which detects a phase shift between p- and s-components of polarized light propagating through the waveguide caused by the molecular adsorption. The refractive index sensitivity (RIS) of the recently upgraded PI experimental setup has been improved and reached values of around 9600 rad per refractive index unity (RIU), the highest RIS values reported, which enables the detection of low molecular weight analytes such as mycotoxins in very low concentrations. The biosensing tests yielded remarkable results for the detection of aflatoxin B1 in a wide range of concentrations from 1 pg/mL to 1 μg/mL in direct assay with specific DNA-based aptamers.

**Electronic supplementary material:**

The online version of this article (10.1007/s00216-019-02033-4) contains supplementary material, which is available to authorized users.

**Graphical abstract:**
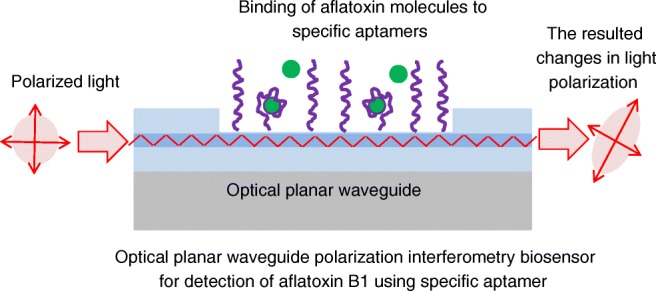
Optical planar waveguide polarization interferometry biosensor for detection of aflatoxin B1 using specific aptamer.

## Introduction

This work is dedicated to development of novel optical biosensing technologies for detection of low molecular weight analytes such as toxins. Our particular target was the detection of mycotoxins being products of metabolism of numerous fungi species which are the most common (particularly in tropical countries) and dangerous contaminants in food and animal feed [1]. Because of high toxic, carcinogenic, and endocrine-disruptive properties of mycotoxins on human, worldwide legislation established quite strict limits on mycotoxin content in food and feed, typically at the ppb (part per billion) concentration level [2]. This makes the detection of small mycotoxin molecules (with typical molecular weight in hundreds of daltons) a difficult task. The development of biosensors for mycotoxins as a cost-effective alternative to the modern advanced analytical methods, such as HPLC and mass spectroscopy, is in great demand nowadays. Such biosensors should be highly sensitive, but at the same time inexpensive, portable, and simple to use to enable quick tests at the point of need.

The detection of mycotoxins is typically done by immunosensing which is based on binding of toxin molecules to specific bioreceptors such as antibodies or aptamers; therefore, optical immunosensing technologies, such as surface plasmon resonance, spectroscopic ellipsometry, optical waveguides, and interferometry, are the most common in mycotoxin detection [3]. The method of total internal reflection ellipsometry (TIRE) was successfully used for detection of several mycotoxins, e.g., T-2, zearalenone, aflatoxin B1, and ochratoxin A, in direct immunoassays with specific antibodies [4–7]. The measurements of phase changes between p- and s-components of polarized light in the method of TIRE provided a high sensitivity of detection of mycotoxins in ppb level of concentration which corresponds well to the legislated limits. However, the method of TIRE is still based on a bulky and expensive optical instrument, e.g., spectroscopic ellipsometer. A logical continuation of this direction in biosensor development was the use of optical planar waveguide (OPW) operating as a polarization interferometer (PI) with the multiperiodic output signal proportional to the phase shift between p- and s-components of polarized light [8]. Previous attempts in this development were encouraging [9, 10]; the PI OPW devices demonstrated high refractive index sensitivity (RIS) which allows the detection of mycotoxins (aflatoxin B1, ochratoxin A, and zearalenone) in concentrations down to 0.01 ppb. During the last couple of years, the PI OPW experimental setup underwent several rounds of upgrading. The latest version, which is based on the waveguide having a Si_3_N_4_ core etched in a narrow (1 mm wide) strip and uses HeNe laser (633 nm) as a light source, offers much higher refractive index sensitivity and much more stable and less noisy output signal.

The use of planar waveguides is, perhaps, the most popular direction nowadays in the development of optical biosensors. Several optical PW biosensor devices developed recently are based on the Mach–Zehnder (MZ) interferometer [11–15] and ring resonator [16, 17] principles; both approaches demonstrated remarkable refractive index sensitivity around 7000 to 8000 rad/refractive index unit (RIU) [11, 17]. The development of fully integrated all-silicon biosensors comprising multichannel MZ interferometers with embedded avalanche LEDs as light sources, sensitive photodetectors, and microfluidic sample delivery system [18–21] was the pinnacle of this direction of research and development; such highly sensitive and portable devices showed their versatility in a wide range of applications from environmental to biomedical, and suitability for in-field or point-of-care detection of analytes of interest. The proposed PI biosensor has similar sensitivity to that of MZ-based devices, but achieved with much simpler design without splitting the waveguide [8–10].

Another idea which we are exploring in this work is the use of aptamers as bioreceptors for mycotoxins. Aptamers being linear biopolymers with specifically designed sequences of RNA or DNA nucleotides to bind target molecules are getting increasingly popular nowadays as artificial bioreceptors in various biosensing applications as alternative to antibodies [22, 23]. Aptamers have a number of advantages over traditional antibodies, mainly in their robustness, simple immobilization chemistry, and lower cost. The technology of aptamer synthesis improved dramatically in the last decade, so they became commercially available for a wide variety of analytes. Aptamers were recently used successfully in detecting mycotoxins, e.g., ochratoxin A [24–26]. In majority of biosensing applications, aptamers are usually labeled with either fluorescent or redox groups enabling the use of simple optical or electrochemical detection, respectively. In our recent work, we successfully tried unlabeled aptamers for ochratoxin A [7] and aflatoxin B1 [27] in conjunction with highly sensitive TIRE detection method. In this work, we explore the use of aptamers as specific bioreceptors for in vitro detection of aflatoxin B1 in a combination with a highly sensitive (after recent upgrades) PI OPW method.

## IP OPW experimental setup

**Fig. 1:**
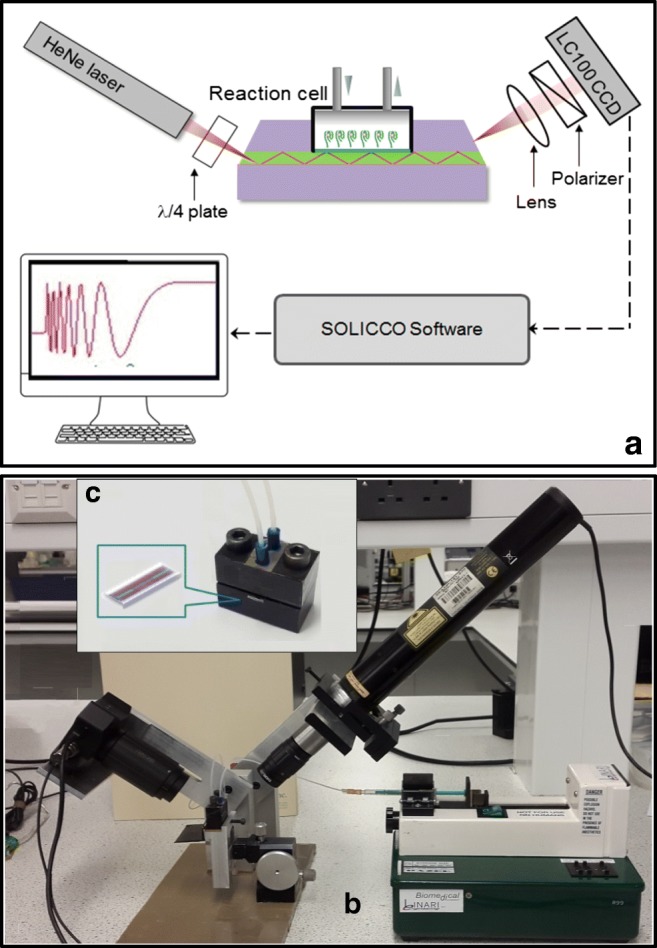
Schematic diagram (**a**) and photograph (**b**) of the PI OPW experimental setup; the reaction cell with inserted OPW (**c**), the inset shows zoomed-in OPW chip

The key element of this system is an optical planar waveguide (shown in Fig. 1) which was produced on silicon wafer using standard microelectronic processes and consisted with the core 200-nm-thick layer of Si_3_N_4_ sandwiched between much thicker (3 μm) layers of SiO_2_. A large difference in refractive indices of the Si_3_N_4_ core (*n* = 2.02) and SiO_2_ cladding (*n* = 1.46) results in a steep angle (47°) of light propagation through the waveguide and thus a large number of reflections which was estimated as 500 per mm of the waveguide length using the Goos–Hanchen model [28]. Using photolithography, the Si_3_N_4_ core layer was formed as a 1-mm-wide strip, and a sensing window was etched in the top SiO_2_ layer (see the image of the OPW chip in Fig. 1c). Etching of the top cladding SiO_2_ layer was carried out in diluted 1:10 hydrofluoric acid (HF), while the S_3_N_4_ core was etched in hot (160 °C) phosphoric acid (H_3_PO_4_). The reaction cell (Fig. 1c) was attached to the sensing window to enable injecting different chemicals and thus performing different biochemical reactions, e.g., binding the analyte molecules to bioreceptors immobilized on the surface of a sensing window.

The light beam from a HeNe laser (*λ* = 632.8 nm) circularly polarized by *λ*/4 plate was coupled into the waveguide via slant edge (polished at 47°). As shown in Fig. 1a, b, the light coming out from the other end of the waveguide goes through the polarizer which converts a phase shift between p- and s-components of polarized light into variations of light intensity. The light is collected by CCD array (Thorlabs LC100–Smart Line Camera) which was interfaced to PC; SPLICCO dedicated software was used to record the output signals. The resulting multiperiodic output signal shown in Fig. 1a can be produced either by variations of the refractive index of the medium or by molecular adsorption.

The modeling of the PI OPW setup was carried out by calculating the phase shift between p- and s-components of polarized light caused by changes in the refractive index of the medium in the waveguide sensing window [29]:

$$ \Delta  {\varphi}_{P,S}=2\arctan \left[\frac{\sqrt{\left({N}^2{\sin}^2\theta -1\right)}}{N\ \sin \theta\ \tan \theta }\ \right], $$

where *N = n*_1_*/n*_2_ is the ratio of the refractive indices of the core and cladding, and *θ* is the angle of propagation. The maximal refractive index sensitivity (RIS) at *θ =* 47° was estimated as 1550 rad/RIU (RIU stands for refractive index unity) for 500 reflections per mm, or 9300 rad/RIU on 6-mm-long sensing window.

**Fig. 2:**
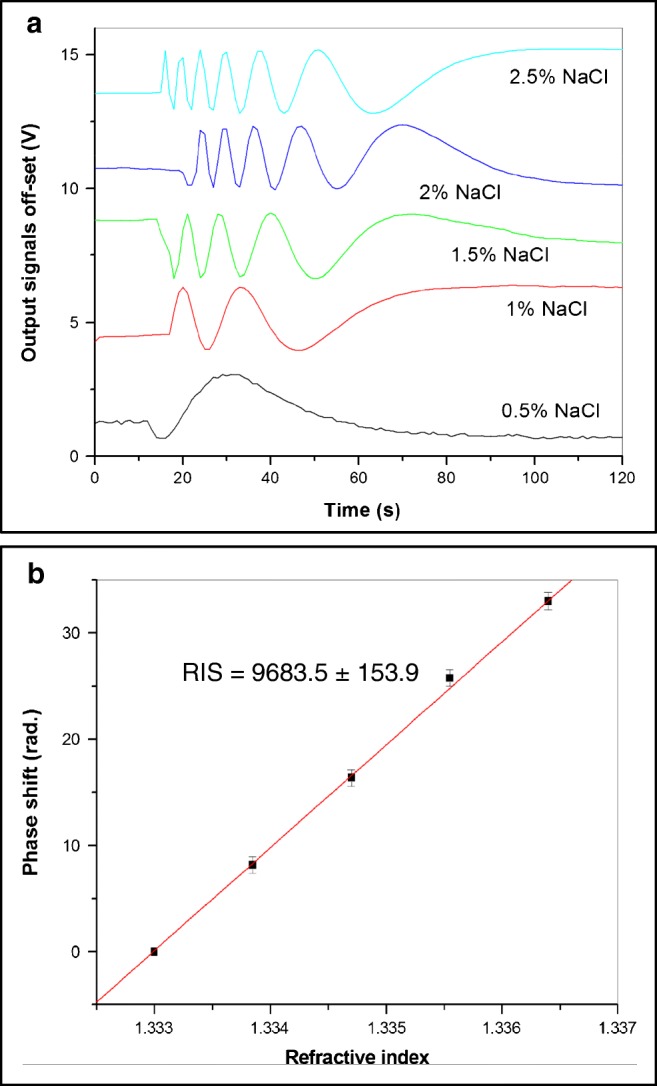
Evaluation of the refractive index sensitivity (RIS): response signals to refractive index changes by injecting NaCl solutions of different concentrations (**a**), the dependence of phase shift against refractive index (**b**)

The refractive index sensitivity (RIS) was assessed experimentally by injecting NaCl aqueous solutions of different concentrations into the cell. Multiperiodic output signals caused by the changes in refractive index were recorded and presented in Fig. 2a, and the number of periods of signal oscillations was precisely calculated from the waveforms and plotted against the refractive index in Fig. 2b. The results of a single typical set of measurements were presented in Fig. 2; the accuracy of phase shift evaluation was estimated as 0.1 rad. The RIS value of 9683.5 ± 153.9 rad/RIU was found as a gradient of the linear dependence in Fig. 2b. The obtained value is close to the estimated RIS of 9300 rad/RIU, and it is higher than the RIS values reported for the other OPW-based sensors [11, 17].

## Preparation of aptamers and their immobilization on OPW

The anti-aflatoxin B1 aptamers were synthesized and purified by Microsynth (Schutzenstrasse, Balgach, Switzerland). The sequence of nucleotides established previously in [30] is shown below:

SH- 5′-GTTGGGCACGTGTTGTCTCTCTGTGTCTCGTGCCCTTCGCTAGGCCCACA-3′

There was no label attached at 3′ (e.g., C3) terminal, while the thiol group was attached to 5′ (e.g., C5) terminal. This aptamer is highly specific to aflatoxin B1 target with the affinity coefficient in the range of 10^−7^ Mol [30] which was independently confirmed in our previous research [27]. The aptamers dissolved in phosphate-binding buffer (PBB) containing MgCl_2_ salt were immobilized on the Si_3_N_4_ surface of sensing window of OPW, which was firstly aminated in (3-aminopropyl)triethoxysilane, via an intermediate layer of 4-(*N*-maleimidomethyl) cyclohexanecarboxylic acid (SMCC).

The aptamers were delivered in lyophilized form. The aptamer stock solution is prepared at 100 μM by adding an appropriate volume of sterilized water. Then, the stock solution is aliquoted and stored at − 20 °C. All other chemicals, e.g., sodium phosphate di-basic (Na_2_HPO_4_), potassium phosphate mono-basic (KH_2_PO_4_), potassium chloride (KCl), magnesium chloride (MgCl_2_), dithiothreitol (DTT), sulfo-succinimidyl-4-(*N*-maleimidomethyl) cyclohexane-1-carboxylate (sulfo-SMCC), and sodium chloride (NaCl), were procured from Sigma-Aldrich (France). Aflatoxin B1 was purchased from Sigma-Aldrich (UK). All reagents were of analytical grade. Deionized Milli-Q water was used for preparation of reagents throughout the experiments. The 100 mM PBS-binding buffer (PBB) was prepared by dissolving 10 mM Na_2_HPO_4_, 1.76 mM KH_2_PO_4_, 3 mM MgCl_2_, 2.7 mM KCl, and 137 mM NaCl in deionized water. The pH value of the buffer was adjusted to 7.4.

**Fig. 3:**
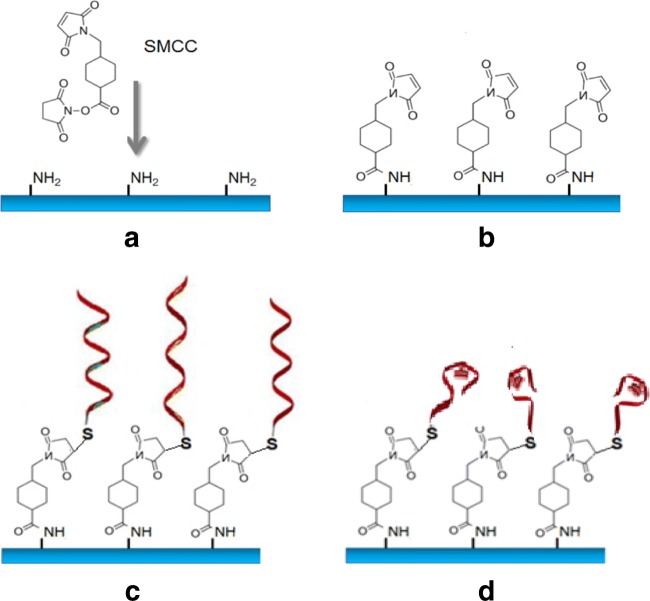
Aptamer immobilization protocol: amine-functionalized surface of Si_3_N_4_ (**a**), SMCC-activated surface (**b**), aptamers immobilized (**c**), and aptamer binding target analyte molecules (**d**)

Before immobilization, the stock solution of aptamers was diluted at desired concentration with PBB supplemented with 2 mM of DTT, then diluted aptamer solution was thermo-cycled in PCR unit, e.g., heated to 90 °C for 5 min and cooled down to 4 °C for 5 min. The presence of thiol groups at 5′ terminal allows immobilization on gold surface. In our case, however, we need to immobilize this aptamer in the sensing window of the planar waveguide on the surface Si_3_Ni_4_. The process of aptamer immobilization is illustrated in Fig. 3.

At first, the silicon nitride surface was functionalized with amine groups using (3-aminopropyl) triethoxysilane (Fig. 3a), then 4-(*N*-maleimidomethyl) cyclohexanecarboxylic acid (SMCC) molecules were bound to amine groups (Fig. 3b) by adding 30 μL of 10 mM SMCC solution in PBS buffer, pH 7.4 to each waveguide chip, and incubating the samples for 1 h at room temperature. SMCC is a heterobifunctional cross-linker that contains *N*-hydroxysuccinimide (NHS) ester and maleimide groups which allow covalent conjugation of amine groups on the surface and SH-groups in aptamers. NHS esters react with primary amines at pH 7–9 to form amide bonds while maleimides react with thiol groups at pH 6.5–7.5 to form stable thiol–ether bonds. The samples were then washed with deionized water three times. Finally, aptamers were attached to the SMCC layer using the specific reaction between C5-terminal cysteines and the maleimide-activated surfaces (Fig. 3c). This was achieved by dropping 30 μL of 2 μM aptamer solution (preheated with thermal cycler) in binding buffer pH 7.4 with the addition of 1 mM DTT (dithiothreitol) on the SMCC-activated plates. The samples were incubated for 4 h at room temperature in the dark. After the conjugation reaction was complete, the samples were washed three times for 2 min in binding buffer.

Before immobilization step on the maleimide-activated surface, the aptamer used was denatured by heating/cooling cycle to ensure that the DNA sequence adopt the most favorable secondary structure combining a loop and a linear part (see the secondary aptamer structure as Electronic Supplementary Material (ESM), Fig. S1). However, in the presence of analytes, e.g., aflatoxin B1, the above aptamer engulfs the target (Fig. 3d), which causes changes in the aptamer’s secondary structure stabilized by the presence of MgCl_2_ salt in binding buffer [31]. This process which is associated with changes in the molecular layer thickness can be easily detected by a PI OPW sensor.

## Detection of aflatoxin B1 with a PI OPW sensor in aptamer assay

Aflatoxin B1 (AFT B1) stock solution (1 mg/mL) was prepared in acetonitrile. Further dilutions of AFT B1 were done in PBB. All of the working AFT B1 solutions of 1 pg/mL, 0.01 ng/mL, 0.1 ng/mL, 1 ng/mL, 10 ng/mL, 100 ng/mL, and 1 μg/mL were prepared freshly before use and stored at 4 °C when not in use.

**Fig. 4:**
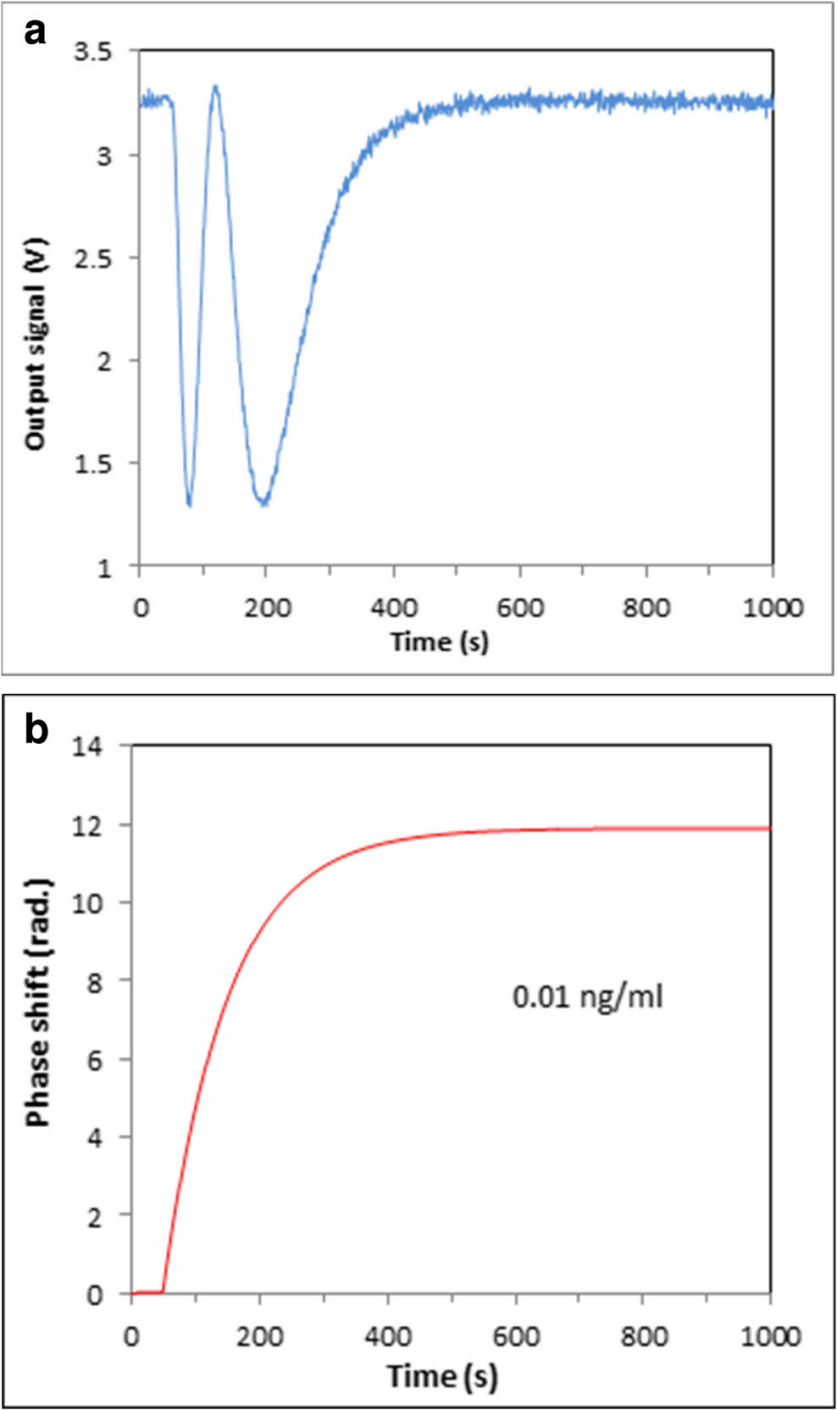
Typical output signal (**a**) and corresponding phase shift (**b**) for injection of AFT_B1_ in concentration of 0.01 ng/mL

A series of PI measurements were carried out by sequential injections of AFT B1 solutions of different concentrations in PBB (starting with the smallest concentration of 1 pg/mL) into the cell. Intermediate washing of the cell by purging it with 1 mL of PBB solution was carried out after each injection in order to remove non-specifically bound toxin molecules. The signals were recorded during binding of AFT B1 to their specific aptamers with the exposure time of around 40 min. The typical recorded waveform for injection of 0.01 ng/mL of AFT B1 is shown in Fig. 4a along with the corresponding phase changes in Fig. 4b. In contrast to our earlier works, where the phase shift was roughly estimated with the accuracy of half-a-period, here the phase shift was calculated more precisely by integration of the waveforms. All results, e.g., the waveforms and corresponding phase shifts, for all concentrations of AFT B1 used are given as ESM in Fig. S2.

**Fig. 5:**
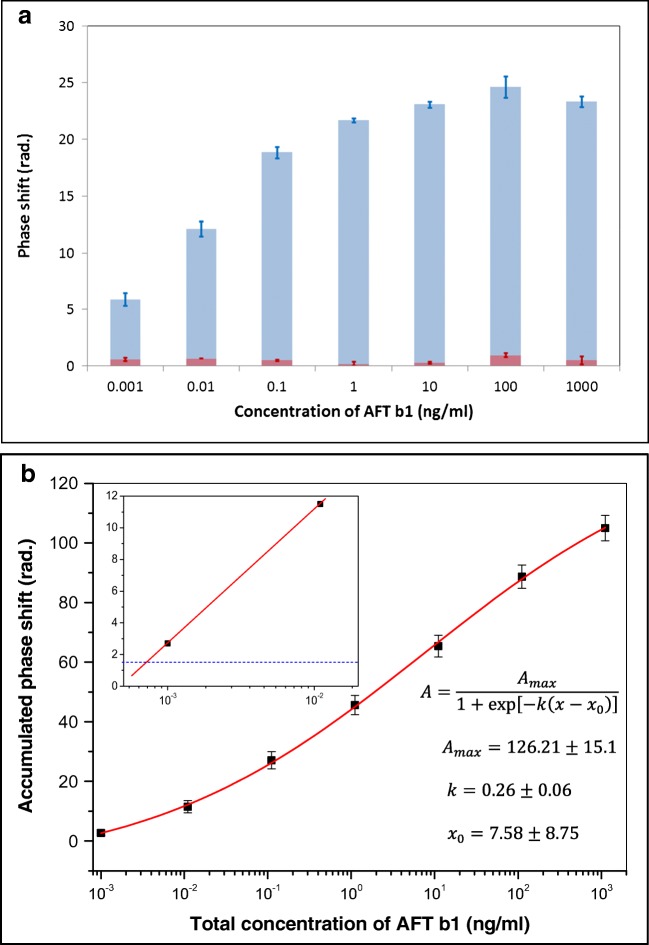
**a** Concentration dependence of phase shifts caused by consecutive injections of AFT B1 (blue columns) and during washing out of toxins (red columns). **b** Accumulated phase shift vs the total concentration of AFT B1. Inset shows zoomed-in section of the diagram (**b**)

As one can see, the lowest concentration of AFT B1 used was 1 pg/mL, which causes a noticeable 5.84 rad (nearly the whole period) of phase change. The saturation of the response typically occurs after 400 s to 500 s of exposure which is similar to the time of immune reaction, e.g., binding target analyte to respective antibodies [9, 10]. The PI OPW sensor responses, e.g., the phase shift values, recorded for consecutive injections of different concentrations of AFT B1 summarized in Fig. 5a (blue columns) show monotonous increase of the phase shift with the increase of AFT B1 concentration. The standard deviation values (shown as error bars) were calculated by statistical analysis of five series of measurements.

A small drop in the response at the highest concentration of 1 μg/mL is most likely caused by saturation of binding sites, e.g. aptamers. The signal recordings, which were performed during washing out non-specifically bound toxin molecules after each binding stage, typically yield 2.36 ± 0.14 rad of phase change (see Fig. 5a, red columns). The fact that this value is practically independent on the AFT B1 concentration indicates that non-specific adsorption most likely takes place in voids between immobilized aptamers. Negative control tests were also carried out by injecting a different mycotoxin, e.g. ochratoxin A, which is not supposed to be bound to the anti-AFT B1 aptamer. The response was at the noise level. Also, the injection of pure buffer solution (with no AFT B1 added) yielded a zero response. These results are shown as ESM in Fig. S3.

Because the measurements were carried out in sequence of steps of injections of toxins in progressively increased concentration, further analysis of the data obtained was carried out in order to work out the accumulated response. For that purpose, the phase changes at each stage were added, and the responses to washing out toxins were subtracted. The resulting calibration curve in Fig. 5b was obtained by fitting the data to sigmoidal (logistic) function using Origin 6.0 software; the formula and fitting parameters are shown in Fig. 5b.

Figure 5b yields a wide linear dynamic detection range (from 0.01 to100 ng/mL) with the trend of flattening at both low and high concentrations. The mean standard deviation of phase shift measurements was estimated as ± 0.5 rad which allows the evaluation of low detection limit as 0.7 pg/mL by linear approximation of calibration curve to the level of 0.5 × 3 = 1.5 rad (see the inset in Fig. 5b).

The results obtained are similar to those reported earlier for the PI detection of aflatoxin B1 in direct immunoassay with specific antibodies [9]. However, the latest upgrades in the PI OPW experimental setup and signal processing in combination with aptamer bioreceptors are clearly more sensitive and thus capable of detection of much smaller concentrations of aflatoxins in sub-ppt range. An additional advantage of using aptamers was a simple procedure of sensor recovery. The OPW sensor chips with immobilized aptamers can be restored to its initial state after performing a heating/cooling cycle in PCR unit (which unravels aptamers and releases the toxin) followed by washing out the toxins in PBB buffer containing MgCl_2_. The recovered OPW chips could be used again after this treatment (such attempts were carried out with the re-used OPW chips showing similar responses); alternatively, the OPW chips immersed in PBB could be stored in a fridge at 4 °C for few weeks. The repeating of a thermo-cycle is advisable before the use.

## Conclusions and future work

The main aim of developing the experimental setup for polarization interferometry biosensor based on optical planar waveguide suitable for in vitro detection of mycotoxins was achieved. After several stages of upgrading, which involved changes in the waveguide design and optical components as well as the improvement of signal processing, the sensor showed much clear (less noisy) output signals with leveled amplitudes. Much higher refractive index sensitivity of around 9600 rad/RIU was achieved as a result, which was similar to the values reported for MZ interferometer-based biosensors [11, 17]. A series of in vitro biosensing tests of detecting aflatoxin B1 in a direct assay with specific aptamer were successful; the PI OPW biosensor was capable of detecting 1 pg/mL of aflatoxin B1. The LDL estimated as 0.7 pg/mL is much lower than that reported for other biosensing techniques, for example, optical methods of SPR (1.5 ng/mL), LSPR using functionalized gold nanoparticles (0.2 ng/mL), LSPR/TIRE (0.01 ng/mL), and electrochemical method of impedance spectroscopy 0.5 ng/mL SPR [32].

The use of aptamers showed several advantages over traditional bioreceptors, e.g., antibodies including the following: (i) the low cost of aptamers, (ii) their stability and re-usability, (iii) relative simplicity of immobilization, and (iv) selectivity similar to that of antibodies. In terms of performance, the PI OPW biosensors are similar to MZ interferometer–based ones, though having a much simpler design. The developed PI OPW biosensors can be used as a platform biosensing technology for detection of various analytes of interest for different applications including environmental control, agriculture and food industry, and biomedical fields.

As a result of the 3-year project funded by NATO SPS, the PI OPW biosensor reached the stage of commercial development. The future work could involve (i) further improvement of the planar waveguide sensor design using photolithography to make several narrow waveguide channels for simultaneous detection of several analytes, (ii) scaling down the sensor design to a hand-held type of devices suitable for the point-of-need biosensing, (iii) further development of the data acquisition system by simultaneous calculation of the phase shift, and (iv) the detection of mycotoxins in a complex matrix of real samples of food extracts and drinks.

## Electronic supplementary material

ESM 1 (PDF 222 kb)
