# Supplementary material for: Highly sensitive label-free in vitro detection of aflatoxin B1 in an aptamer assay using optical planar waveguide operating as a polarization interferometer
Source: Anal Bioanal Chem. 2019 Aug 7;411(29):7717–24. doi: 10.1007/s00216-019-02033-4 (PMC6881424; doi:10.1007/s00216-019-02033-4)
Supplement: Supplementary file 1 — (PDF 222 kb) [file 216_2019_2033_MOESM1_ESM.pdf]

Analytical and Bioanalytical Chemistry

Electronic Supplementary Material

Highly sensitive label-free in vitro detection of aflatoxin B1 in an aptamer assay using an optical planar waveguide operating as a polarization interferometer

Ali Al-Jawdah, Alexei Nabok, Hisham Abu-Ali, Gaelle Catanante, Jean-Louis Marty, Andras Szekacs

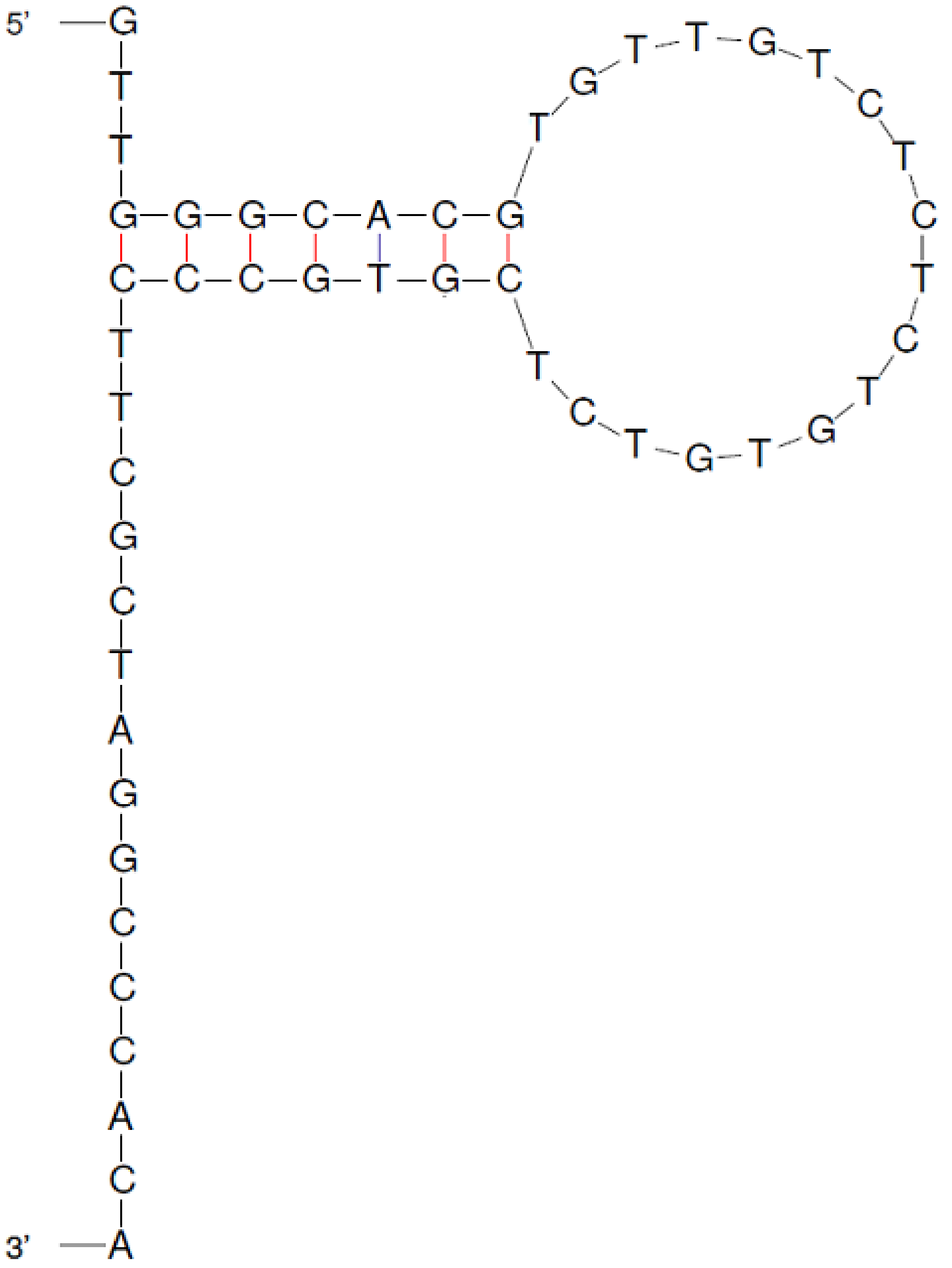

Fig. S1 Secondary structure of anti-aflatoxin B1 aptamer

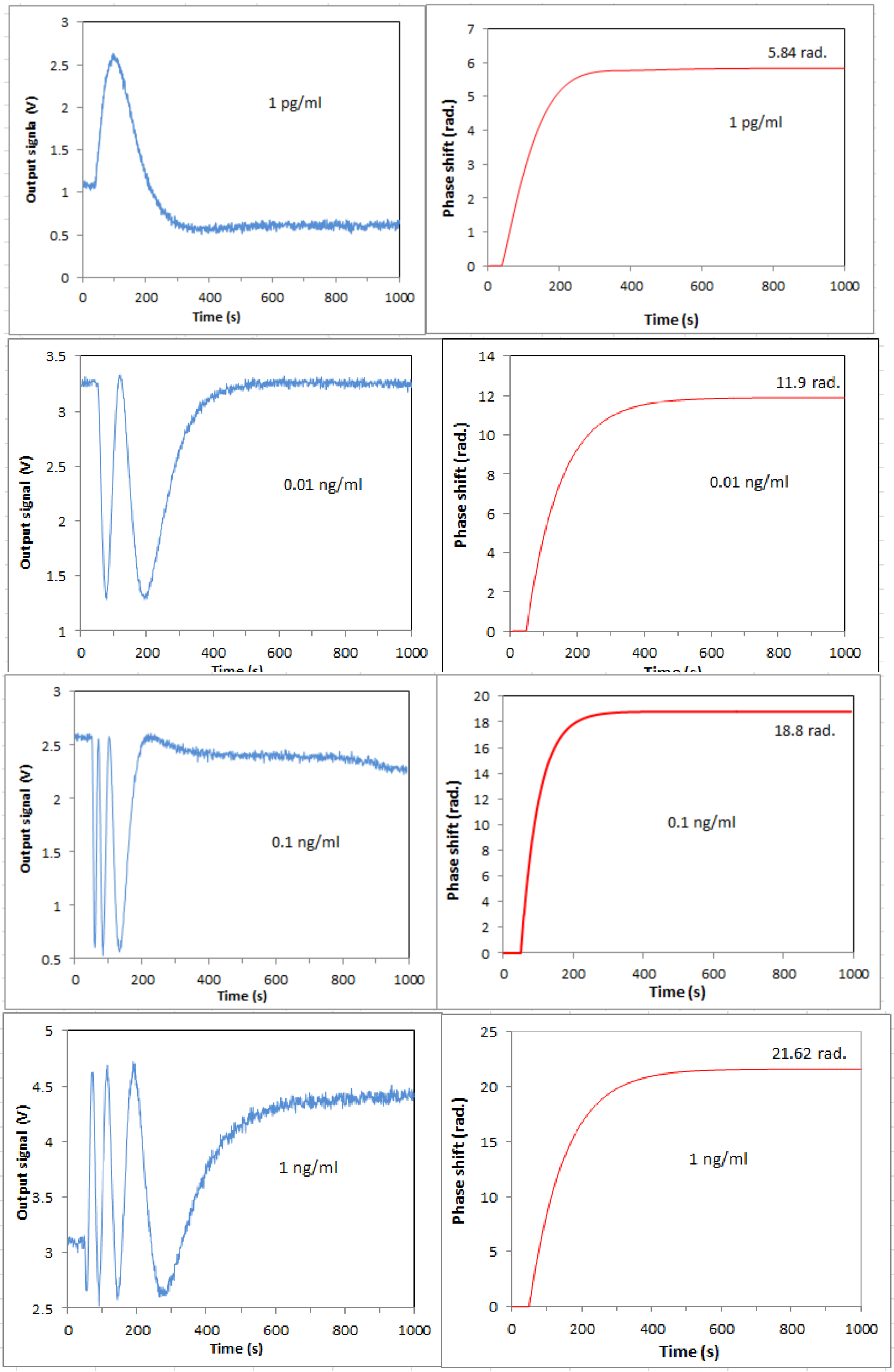
Output signal (V)
Time (s)
1 pg/ml
Phase shift (rad.)
5.84 rad.
0.01 ng/ml
11.9 rad.
0.1 ng/ml
18.8 rad.
1 ng/ml
21.62 rad.

(a)

(b)

(c)

(d)

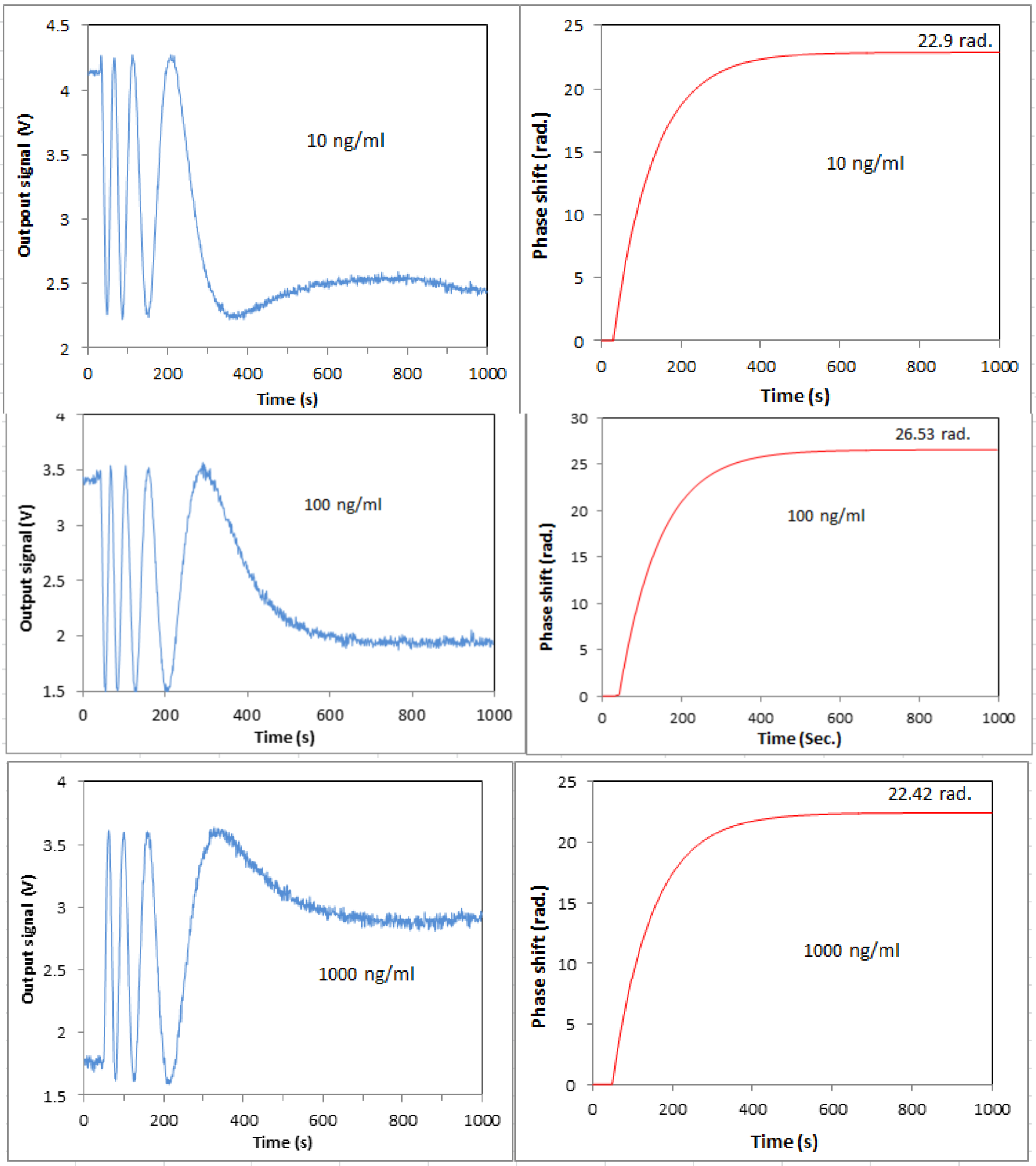

(e)

(f)

(g)

Fig. S2 Output signals (left) and corresponding phase shifts (right) for different concentrations of AFT b1: 0.001 ng/ml (a), 0.01 ng/ml (b), 0.1 ng/ml (c), 1 ng/ml (d), 10 ng/ml (e), 100 ng/ml (f), and 1000 ng/ml (g)

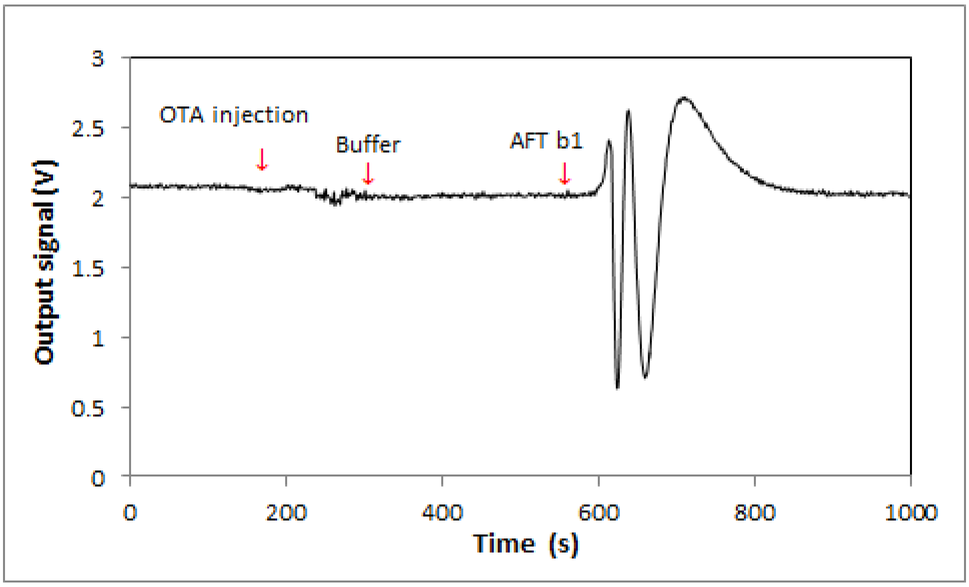

Fig. S3 Negative control data. Output signals caused by injections of OTA (0.01ng/ml) and AFT b1 (0.01 ng/ml). Arrows indicate the moments of injection
